# Microbiota-Mediated Crosstalk Between the Gut and the Vascular System: Protective Effects of Novel Postbiotic Formulations on Human Endothelial and Vascular Smooth Muscle Cells

**DOI:** 10.3390/ijms27021011

**Published:** 2026-01-20

**Authors:** Lorenzo Flori, Diletta Francesca Squarzanti, Marta Lo Re, Patrizia Malfa, Alma Martelli, Vincenzo Calderone

**Affiliations:** 1 Department of Pharmacy, University of Pisa, 56126 Pisa, Italy; alma.martelli@unipi.it (A.M.); vincenzo.calderone@unipi.it (V.C.); 2 R&D, SynBalance SRL, 21040 Origgio, Italy; d.squarzanti@synbalance.care (D.F.S.); m.lore@synbalance.care (M.L.R.); p.malfa@synbalance.care (P.M.); 3 Interdepartmental Research Centre Nutraceuticals and Food for Health—NUTRAFOOD, University of Pisa, 56126 Pisa, Italy; 4 CISUP—Centro per l’Integrazione della Strumentazione dell’Università di Pisa, Lungarno Pacinotti 43, 56126 Pisa, Italy

**Keywords:** gut, vascular, probiotic, postbiotic, gut-vascular axis, microbiota, crosstalk, TMAO, dysbiosis, lactate

## Abstract

The close connections between the intestine and distal systems, known as axes, are a growing focus of scientific research; however, the gut–vascular axis, particularly as a target of microbial metabolites, remains underexplored. In this study, three supernatants derived from probiotic formulations composed of *Lactobacillus* and *Bifidobacterium* strains (MIX-1, MIX-2, and MIX-3) were evaluated in counteracting vascular alterations associated with dysbiosis. Human aortic smooth muscle (HASMCs) and endothelial (HAECs) cells were exposed to pro-oxidative (H_2_O_2_) and pro-inflammatory (TMAO) stimuli. Concentrations up to 5–10% (*v*/*v*) were tolerated in both cell lines, with MIX-1 and MIX-3 showing the greatest protective efficacy. These formulations exerted antioxidant effects by reducing H_2_O_2_-induced ROS production and cell viability loss, and anti-inflammatory effects by limiting TMAO-induced IL-1β release. MIX-1 also attenuated TMAO-induced IL-6 release. Further analyses indicated a partial involvement of the SIRT1-pathway in its vascular antioxidant effects. Chromatographic profiling revealed comparable qualitative metabolites among the probiotic supernatants, while quantitative differences were observed, with higher lactate levels in MIX-1 and MIX-3 compared to MIX-2. Finally, we have determined that *Limosilactobacillus reuteri*-PBS072 is mainly responsible for the antioxidant effect of MIX-1 and MIX-3. Overall, these findings highlight the potential of probiotic-derived metabolites in modulating the gut–vascular axis and promoting vascular protection.

## 1. Introduction

The intestine has attracted considerable interest because of its central role in regulating physio-immunopathological processes, earning it the nickname, “the second brain.” This new paradigm therefore considers the intestine as the center of the human organism, capable of influencing distal organs and communicating with them through systemic circulation [1,2].

The gut is colonized by the intestinal microbiota, composed of trillions of non-pathogenic microorganisms, including bacteria, viruses, fungi, and archaea, forming a complex and dynamic ecosystem that plays a crucial role in maintaining the body’s health [3]. The gut microbiota contributes to the maintenance of homeostasis by preserving the integrity of the intestinal barrier, modulating inflammatory processes, and regulating permeability to pathogens. It modulates immune responses and influences drug metabolism. Furthermore, it acts as an endocrine organ, producing metabolites that can be absorbed into the bloodstream through the intestinal epithelium and exert effects on distant organs, promoting their physiological functions and modulating the progression of pathological changes [4,5,6].

Alterations in the gut microbiota composition (dysbiosis) compromise metabolic homeostasis and the integrity of the intestinal barrier, increasing intestinal permeability and defining a condition known as “leaky gut.” This condition, typical of inflammatory bowel diseases, is associated with marked imbalances in microbial composition, such as alterations in the *Bacteroidetes*/*Firmicutes* ratio, and with inflammatory activation mediated by lipopolysaccharide (LPS), trimethylamine N-oxide (TMAO), and pro-inflammatory cytokines [7].

Moreover, alterations in the gut microbiota are closely linked to pathological scenarios in peripheral tissues. Among these, dysbiosis contributes to the development of vascular diseases, such as atherosclerosis, hypertension, and endothelial dysfunction, through the disruption of specific bacterial populations and the consequent altered production of metabolites [8,9]. This pathophysiological link between the gut microbiota and blood vessels clearly demonstrates the existence of a gut–vascular axis: blood vessels, once regarded merely as a simple “road network” traversed by intestinal metabolites on their way to target organs (for instance, the liver, kidney, brain and hearth), are now recognized as a true target organ in their own right upon which these metabolites act [10,11,12,13]. To date, however, the crosstalk between the microbiota and blood vessels, as a target of metabolites, remains underestimated, despite the fact that vascular pathophysiology is strongly influenced by intestinal microbiota-derived metabolites and that several vascular pathologies are associated with microbiota alterations [14]. In this regard, experimental evidence suggests a common pattern in vascular diseases, indicating that dysbiosis can promote endothelial dysfunction and hypertension through increased levels of pro-inflammatory and pro-oxidant metabolites, such as TMAO, along with a concomitant rise in reactive oxygen species (ROS) [15,16,17]. Therefore, it appears evident that dysbiotic alterations in microbiota-derived metabolites contribute to damage of the gut–vascular axis. With the aim of restoring intestinal metabolic homeostasis, a well-established approach involves the use of probiotics, which have been shown to confer benefits for cardiovascular health [18]. In endothelial cells, specific probiotic strains have been shown to enhance nitric oxide (NO) bioavailability, reduce oxidative stress, and attenuate pro-inflammatory signaling pathways, thereby preserving endothelial homeostasis. In parallel, probiotics and microbiota-derived metabolites have been reported to influence vascular smooth muscle cells by modulating redox balance, contractile responses, and proliferation, ultimately affecting vascular tone and remodeling [19,20].

Furthermore, an innovative approach of considerable interest is represented by postbiotics, defined as preparations of intact inanimate microorganisms and/or their fragments or metabolites capable of conferring health benefits to the host [21]. Postbiotics can interact with the gut–vascular axis by restoring intestinal homeostasis and epithelial barrier integrity, as well as by acting directly on the vascular district [10]. Although preclinical studies suggest beneficial effects of individual metabolites or specific combinations in reducing inflammation, oxidative stress, and endothelial dysfunction, the impact of complex mixtures of metabolites derived from bacterial strains on vascular health in the presence of pathological conditions has not yet been thoroughly investigated [22]. Further characterization of the beneficial effects of probiotic formulations on the vascular system could pave the way for their use, either as such or as postbiotic combinations, capable of providing a dual beneficial effect: restoring intestinal dysbiosis and generating a broad spectrum of metabolites in situ, with activity along the gut–vascular axis.

In the present study, three postbiotic formulations derived from distinct probiotic combinations were selected to investigate their potential role in protecting vascular function under conditions of oxidative and inflammatory stress associated with dysbiosis. These formulations include bacterial strains belonging to the *Lactobacillus* and *Bifidobacterium* genera, which are recognized for their capacity to generate bioactive metabolites capable of modulating host redox homeostasis and inflammatory pathways. Notably, strains of the *Lactobacillus* genus, which prevail in the formulations examined, have been already partially investigated for their beneficial effects on vascular health, including the attenuation of endothelial dysfunction, oxidative stress, and low-grade inflammation, all hallmarks of dysbiosis-related vascular impairment [23,24,25,26].

From this perspective, the present work aims to comparatively evaluate the efficacy of postbiotic preparations derived from these three defined probiotic formulations in mitigating dysbiosis-related oxidative and inflammatory vascular damage, to characterize their qualitative and quantitative metabolic compositions, and preliminarily investigate the involvement of specific molecular pathways, thereby providing insight into their potential relevance for vascular protection.

## 2. Results

### 2.1. Qualitative/Quantitative Characterization of Postbiotic Formulations

**Figure 1:**
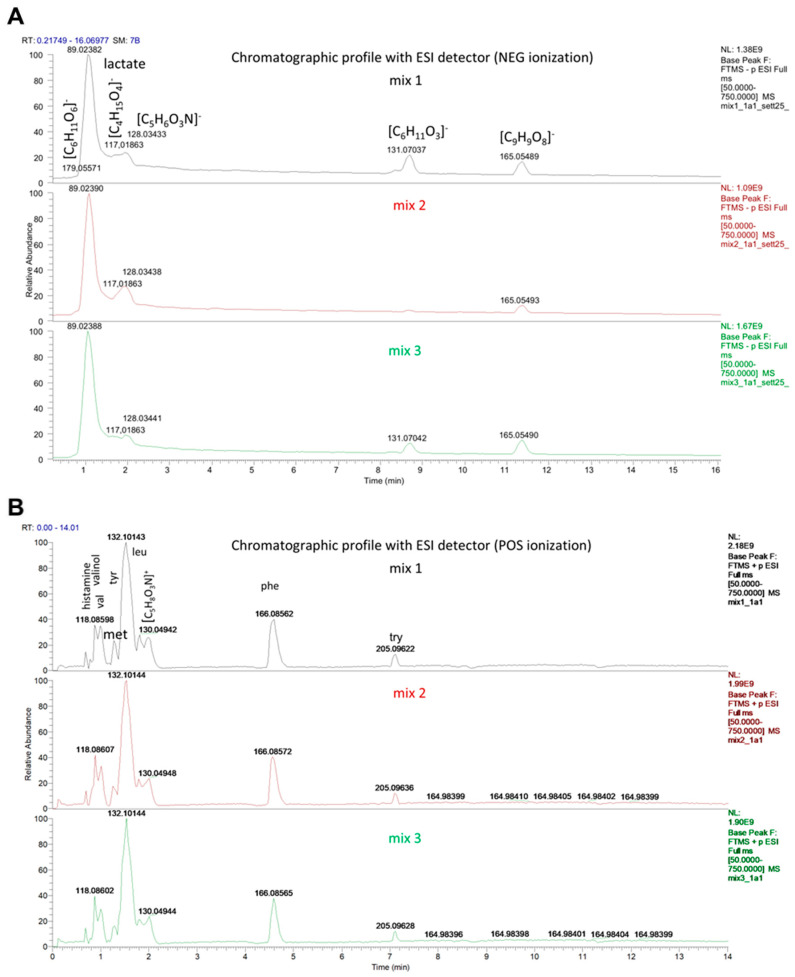
C-HRMS profiles of MIX-1 (black), MIX-2 (red), and MIX-3 (green) recorded in negative ESI mode (**A**). LC-HRMS profiles of MIX-1, MIX-2, and MIX-3 recorded in positive ESI mode (**B**).

The chromatographic profiles of the metabolites contained in the 3 postbiotic formulations (MIXes) showed a high degree of qualitative overlap, indicating a comparable overall composition (Figure 1A,B). Nevertheless, quantitative analysis revealed significant differences and clear trends in the relative abundance of specific metabolites. In particular, MIX-1 (4.04 mg/mL ± 0.24) showed higher lactate levels, followed by MIX-3 (3.35 mg/mL ± 0.31) and finally MIX-2 (2.71 mg/mL ± 0.15) (Table 1).

### 2.2. Safety Profile

**Figure 2:**
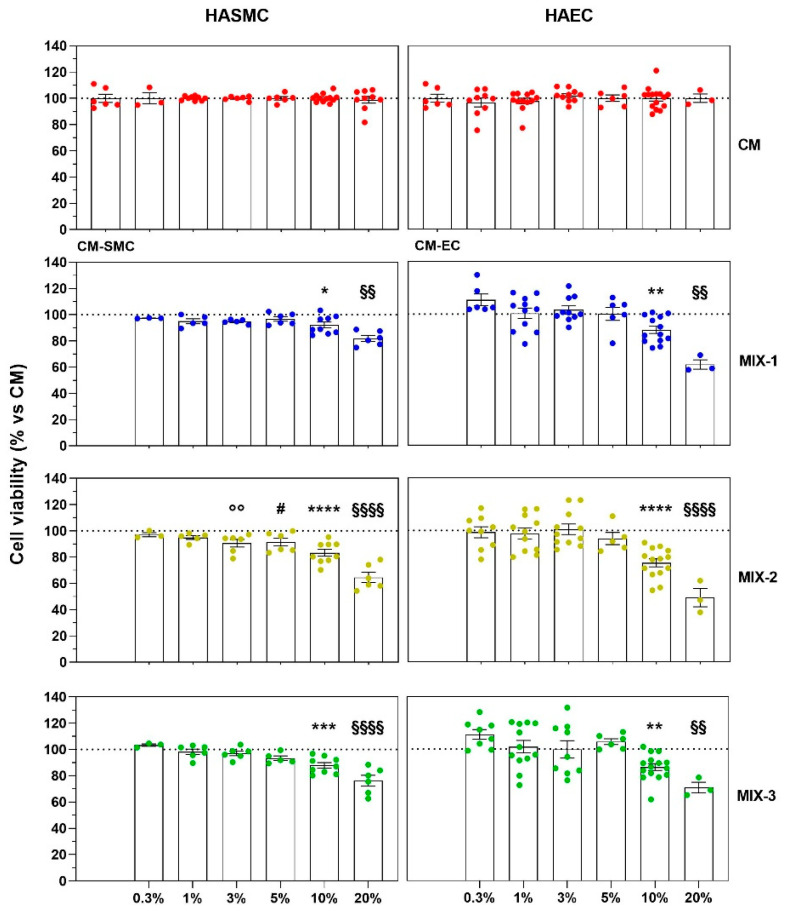
The vertical left graphs of the plots grid represent the results of cell viability on vascular smooth muscle cells (HASMCs), while the vertical right graphs column represents the results on human aortic endothelial cells (HAECs). The horizontal graphs of the plots grid represent the cytotoxicity at different concentrations (0.3%, 1%, 3%, 5%, 10%, 20%) of Vehicle (High Glucose-HG, red dot points), MIX-1 (blue dot points), MIX-2 (yellow dot points), or MIX-3 (green dot points) (from top to bottom). Cell viability was expressed normalizing as a percentage of culture medium of smooth muscle cells (CM-SMC) or culture medium of endothelial cells (CM-EC), identified as 100% cell viability (dash lines). Statistical significance versus HG 3% (°° *p* < 0.01), versus HG 5% (# *p* < 0.05), versus HG 10% (* *p* < 0.05; ** *p* < 0.01; *** *p* < 0.001; **** *p* < 0.0001), versus HG 20% (§§ *p* < 0.01; §§§§ *p* < 0.0001). The minimum number of replicates for each treatment was 9. Where the number of replicates differs from that indicated, data points were excluded based on outlier testing. The specific number of replicates considered in the statistical analysis is indicated by the dot points.

The complete absence of cytotoxicity of the HG medium, used as a vehicle in all preclinical experimental procedures, was demonstrated in both cell lines employed (HASMC and HAEC) at concentrations of up to 20% (Figure 2). With regard to safety, all three MIXes exhibited an overall satisfactory profile in both HASMC and HAEC cell lines. In particular, MIX-2 began to show a very slow reduction in cell viability in HASMCs starting at a concentration of 3%, whereas in HAECs it remained non-cytotoxic at concentrations up to 10%. Slight but statistically significant cytotoxic effects of MIX-1 and MIX-3 in both cell lines were observed only at 10%. At the highest concentration tested (20%), all three MIXes displayed a further, concentration-dependent increase in cellular toxicity (Figure 2).

### 2.3. Cell Viability After Pro-Oxidative Stimulation

**Figure 3:**
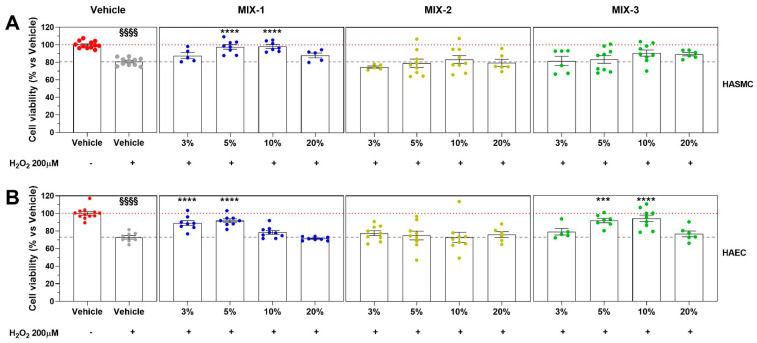
Cell viability after pro-oxidative stimuli (H_2_O_2_ 200 μM on HASMCs and HAECs) expressed normalizing as a percentage of Vehicle alone (HG 20%, red dot points; identified as 100% cell viability, red dash line). The vertical graphs of the plots grid represent the results of H_2_O_2_ (gray dot points and gray dash line), MIX-1 (blue dot points), MIX-2 (yellow dot points), or MIX-3 (green dot points) (from left to right). The horizontal graphs of the plots grid represent the cytotoxicity at different concentrations (3%, 5%, 10%, 20%) on HASMCs (**A**) and HAECs (**B**). Statistical significance versus Vehicle (HG 20%—§§§§ *p* < 0.0001), versus Vehicle (HG 20% + H_2_O_2_—*** *p* < 0.001; **** *p* < 0.0001). The minimum number of replicates for each treatment was 9. Where the number of replicates differs from that indicated, data points were excluded based on outlier testing. The specific number of replicates considered in the statistical analysis is indicated by the dot points.

The results obtained following pre-incubation of HASMCs with the three MIXes at the concentrations selected on the basis of their cytotoxicity profile, followed by exposure to H_2_O_2,_ showed that MIX-1 was able to significantly attenuate the decline in cell viability at concentrations of 5% and 10%. In contrast, at concentrations of 3% and 20%, MIX-1 did not provide a significant protective effect. MIX-2 did not exhibit efficacy in limiting the cellular damage induced by the pro-oxidative stimulus, whereas MIX-3, although not reaching statistical significance, displayed a positive trend at the two highest concentrations tested (10% and 20%) (Figure 3A). A similar evaluation performed in HAECs confirmed a greater protective effect of MIX-1, which significantly attenuated the decline in cell viability at concentrations of 3% and 5%. MIX-3 showed improved efficacy at concentrations of 5% and 10%. Consistent with the findings in HASMCs, MIX-2 did not demonstrate significant efficacy in limiting H_2_O_2_-induced cellular damage (Figure 3B).

### 2.4. ROS Production After Pro-Oxidative Stimulation

**Figure 4:**
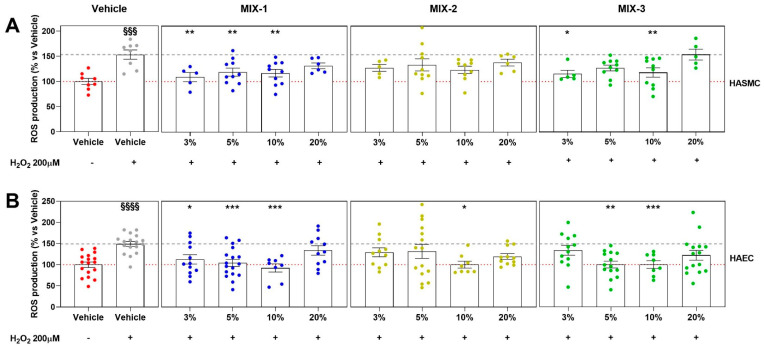
ROS production after pro-oxidative stimuli (H_2_O_2_ 200 μM on HASMCs and HAECs) expressed normalizing as a percentage of vehicle alone (HG 20%, red dot points; identified as 100% ROS production, red dash line). The vertical graphs of the plots grid represent the results of H_2_O_2_ (gray dot points and gray dash line), MIX-1 (blue dot points), MIX-2 (yellow dot points), or MIX-3 (green dot points) (from left to right). The horizontal graphs of the plots grid represent the ROS production at different concentrations (3%, 5%, 10%, 20%) on HASMCs (**A**) and HAECs (**B**). Statistical significance versus Vehicle (HG 20%—§§§ *p* < 0.001; §§§§ *p* < 0.0001), versus Vehicle (HG 20% + H_2_O_2_—* *p* < 0.05; ** *p* < 0.01; *** *p* < 0.001). The minimum number of replicates for each treatment was 9. Where the number of replicates differs from that indicated, data points were excluded based on outlier testing. The specific number of replicates considered in the statistical analysis is indicated by the dot points.

Assessment of ROS production following pro-oxidative stimulation with H_2_O_2_ in HASMCs revealed greater efficacy of MIX-1, which significantly attenuated ROS generation at concentrations of 3%, 5%, and 10%. MIX-3 also significantly reduced ROS production at concentrations of 3% and 10%. MIX-2, although not reaching statistical significance, showed a positive trend in the containment of ROS production (Figure 4A). A similar evaluation performed in HAECs confirmed a statistically significant reduction in ROS production by MIX-1 at concentrations of 3%, 5%, and 10%; MIX-3 significantly attenuated ROS generation at concentrations of 5% and 10%, whereas MIX-2 achieved a statistically significant effect only at the 10% concentration (Figure 4B).

**Figure 5:**
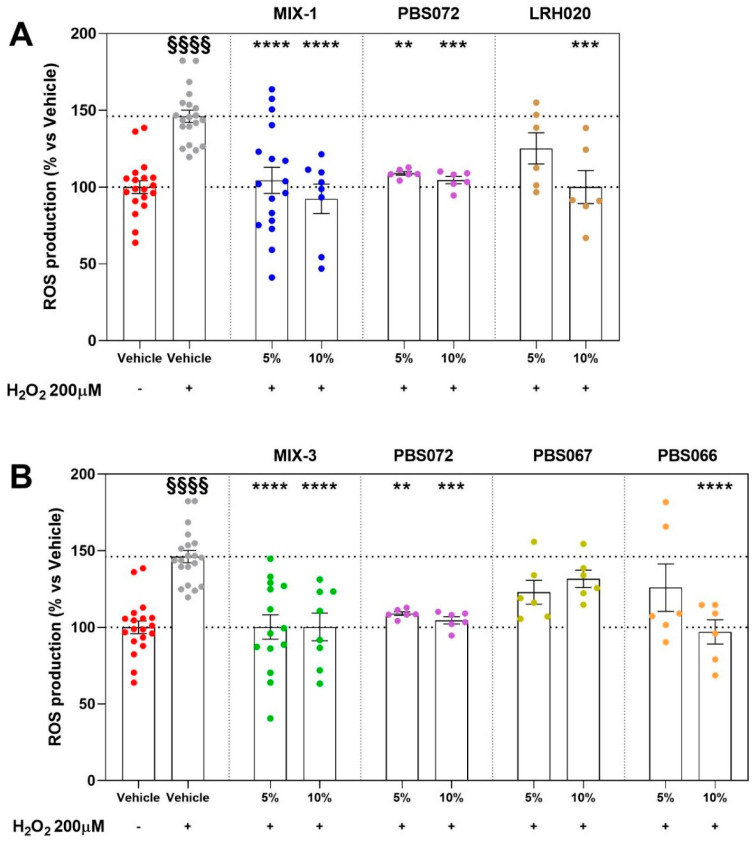
ROS production after pro-oxidative stimulus (H_2_O_2_ 200 μM on HAECs) expressed normalizing as a percentage of vehicle alone (HG 10%; identified as 100% ROS production, lower dash line). Evaluations of the effect from the single bacterial strains (PBS072, PBS067, PBS066, LRH020) at different concentrations (5% and 10%) on HAECs. Upper dash line represents the results of H_2_O_2_. MIX-1 and its strains (**A**); MIX-3 and its strains (**B**). Statistical significance versus Vehicle (HG 10%—§§§§ *p* < 0.0001), versus Vehicle (HG 10% + H_2_O_2_—** *p* < 0.01; *** *p* < 0.001; **** *p* < 0.0001). The minimum number of replicates for each treatment was 6. Where the number of replicates differs from that indicated, data points were excluded based on outlier testing. The specific number of replicates considered in the statistical analysis is indicated by the dot points.

Analyses of the supernatants from individual *Lactobacillus* strains included in the two most effective formulations, MIX-1 (*Lacticaseibacillus rhamnosus* LRH020, *Limosilactobacillus reuteri* PBS072) and MIX-3 (*Lactiplantibacillus plantarum* PBS067, *Lactobacillus acidophilus* PBS066, *Limosilactobacillus reuteri* PBS072), demonstrated distinct strain-specific antioxidant profiles in HAECs. Interestingly, *L. reuteri* PBS072, the strain common to both formulations, consistently showed the strongest activity, significantly reducing ROS production at both tested concentrations (5% and 10%). About MIX-1, *L. rhamnosus* LRH020 produced a significant reduction in ROS levels, although only at the 10% concentration (Figure 5A). In MIX-3, *L. acidophilus* PBS066 elicited a significant decrease in ROS production at the 10% concentration, whereas *L. plantarum* PBS067 did not produce any significant effect at either concentration (Figure 5B).

### 2.5. Anti-Inflammatory Profile

**Figure 6:**
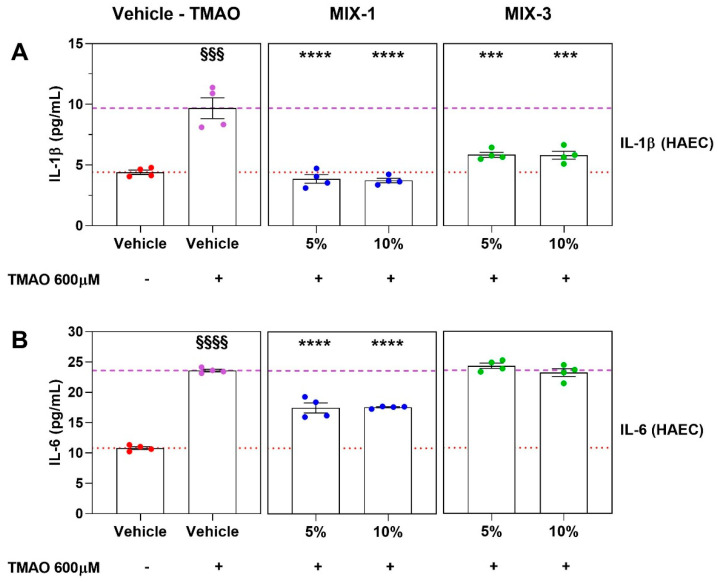
Production of pro-inflammatory cytokines (IL-1β and IL-6) after pro-inflammatory stimuli (TMAO 600 μM) expressed in pg/mL. Lower dash line represents the results of HG 10%. Upper dash line represents the results of TMAO. Concentrations of IL-1β (**A**) and of IL-6 (**B**) at different supernatant concentrations (5%, 10%) on HAECs of MIX-1 (blue dot points) and MIX-3 (green dot points). Statistical significance versus Vehicle (HG 10%—§§§ *p* < 0.001; §§§§ *p* < 0.0001), versus Vehicle (HG 10% + TMAO—*** *p* < 0.001; **** *p* < 0.0001). The minimum number of replicates for each treatment was 9. Where the number of replicates differs from that indicated, data points were excluded based on outlier testing. The specific number of replicates considered in the statistical analysis is indicated by the dot points.

Evaluation of the anti-inflammatory potential of MIX-1 and MIX-3 on HAECs, following pre-incubation with TMAO, indicates that MIX-1, at concentrations of 5% and 10%, exhibited marked anti-inflammatory activity, significantly attenuating the TMAO-induced increase in both IL-1β and IL-6 levels, under the experimental conditions used. In contrast, pre-incubation with MIX-3 at concentrations of 5% and 10% resulted in a significant reduction in IL-1β production (Figure 6A), while no significant effect was observed on IL-6 levels (Figure 6B).

### 2.6. Involvement of the Sirtuins Pathway

**Figure 7:**
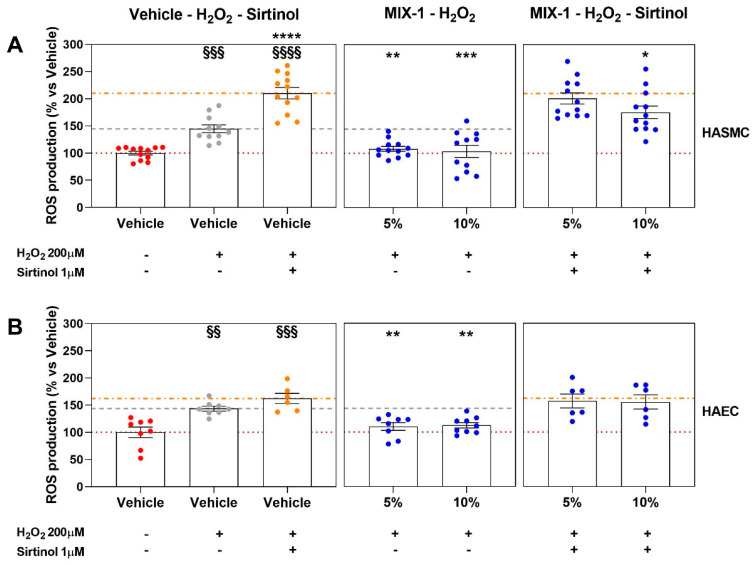
ROS production after pro-oxidative stimuli (H_2_O_2_ 200 μM on HASMCs and 100 μM on HAECs) expressed normalizing as a percentage of vehicle alone (HG 10%, red dot points; identified as 100% ROS production, red dash line). The vertical graphs of the plots grid represent the results of H_2_O_2_ (gray dot points and gray dash line) and H_2_O_2_ + sirtinol (orange dot points and orange dash line), H_2_O_2_ + MIX-1 (blue dot points), H_2_O_2_ + sirtinol + MIX-1 (blue dot points) (from left to right). The horizontal graphs of the plots grid represent the ROS production at different concentrations (5% and 10%) on HASMCs (**A**) and HAECs (**B**). Statistical significance versus Vehicle (HG 10%—§§ *p* < 0.01; §§§ *p* < 0.001; §§§§ *p* < 0.0001), versus Vehicle (HG 10% + H_2_O_2_—* *p* < 0.05; ** *p* < 0.01; *** *p* < 0.001; **** *p* < 0.0001). The minimum number of replicates for each treatment was 9. Where the number of replicates differs from that indicated, data points were excluded based on outlier testing. The specific number of replicates considered in the statistical analysis is indicated by the dot points.

The potential involvement of the sirtuin pathway in the attenuation of ROS production following pro-oxidative stimulation with H_2_O_2_ was evaluated for MIX-1 at concentrations of 5% and 10%, selected as optimal, in both HAEC and HASMC cell lines. Pharmacological inhibition with sirtinol, a selective inhibitor of the SIRT1 and SIRT2 isoforms, significantly increased basal ROS levels compared with both vehicle-treated controls (CM-SMC and CM-EC) and cells exposed to the pro-oxidative stimulus alone in the absence of sirtuin inhibition. Moreover, sirtinol limited (in HASMCs) or completely suppressed (in HAECs) the antioxidant effects observed with MIX-1 at concentrations of 5% and 10% in the absence of sirtinol (Figure 7A and Figure 7B, respectively).

## 3. Discussion

This preclinical experimental study involved the use of in vitro assays on HASMCs and HAECs to closely investigate the effects of three postbiotic formulations, derived from probiotic strains belonging to different genera (*Lactobacillus* and *Bifidobacterium*). The integrity and functionality of the vascular component were investigated to better understand its crosstalk with the intestine, especially under dysbiotic conditions, thereby supporting the concept of gut–vascular axis.

Growing evidence on the gut–vascular axis highlights that microbial metabolites are not merely intestinal by-products but active mediators capable of influencing vascular immuno-pathophysiology, thus opening new therapeutic perspectives [27]. In this context, dysbiosis appears to act as a causal driver of vascular alterations rather than as a simple comorbidity associated with vascular disease [28]. Dysbiosis promotes a pro-inflammatory and pro-oxidative background that accelerates endothelial dysfunction, hypertension, and atherosclerosis. Since the gut microbiota deeply regulates immune responses, metabolic pathways, and epithelial barrier integrity, its preservation emerges as a crucial determinant of vascular and systemic health [29,30]. Consistent with this view, the use of probiotics and prebiotics has been shown to exert beneficial effects on blood pressure regulation and vascular inflammation, supporting the concept that microbiota-targeted interventions may complement standard cardiovascular therapies [18,31,32]. Beyond these approaches, postbiotics represent an innovative and promising strategy, combining the ability to restore intestinal homeostasis with direct effects on the vascular district, potentially overcoming some of the limitations associated with live microbial interventions, including improved formulation flexibility, greater stability, enhanced safety, and suitability for vulnerable populations [33,34,35].

Our results confirmed the potential efficacy of postbiotic formulations on the vascular component. Specifically, the tested supernatants showed varying degrees of efficacy, suggesting that some metabolites, their combinations, quantitative differences, or the derivation from specific bacterial strains belonging to the genus *Lactobacillus* may contribute more than other strains to the restoration of vascular health. The results also suggest a different potency, promoting positive effects at different concentrations. Specifically, MIX-1 emerged as the most promising across all parameters analyzed, followed by MIX-3, which, albeit less effective, still yielded interesting results, and finally MIX-2, which was characterized by marginal efficacy in the experimental procedures used.

Chromatographic profiling revealed that the three tested postbiotic formulations exhibited highly comparable qualitative compositions, with nearly overlapping patterns. Interestingly, however, MIX-1 exhibited significantly higher lactate levels compared with MIX-2, while MIX-3 showed an intermediate increasing trend. Although lactate levels appeared to be, at least partially, positively related to the superior efficacy of MIX-1 and MIX-3 observed across all experimental assays, the contribution of other metabolites, as well as potential synergistic interactions among them, cannot be excluded and may have played a role in the observed biological effects. Although direct extrapolation from in vitro conditions to in vivo exposure remains challenging, the estimated lactate concentrations achieved using 5–20% postbiotic formulations are consistent with physiologically relevant ranges reported to exert beneficial effects on endothelial function, particularly during transient metabolic fluctuations [36].

MIX-1 showed greater vascular tolerability, with the onset of significant cytotoxicity observed only at the highest concentrations tested (10% and 20%) in both HASMCs and HAECs. MIX-3 showed a toxicity profile comparable to that of MIX-1, whereas MIX-2 appeared to show a slightly reduced tolerability compared to the other postbiotic formulations. For all three formulations tested, cytotoxic effect at 24 h were more pronounced in HAECs than on HASMCs.

Both MIX-1 and MIX-3 were able to exert positive effects against oxidative and inflammatory alterations which, under dysbiotic conditions, negatively affect vascular health by reducing the function of both smooth muscle and endothelial components. Specifically, MIX-1 and MIX-3 inhibited the cytotoxic effect and ROS production promoted by a pro-oxidative stimulation (H_2_O_2_), as well as the production of inflammatory cytokines triggered by the pro-inflammatory stimulus (TMAO), specifically generated during intestinal dysbiosis and leaky gut [37]. Among these two more effective formulations, as previously mentioned, MIX-1 demonstrated greater efficacy and potency than MIX-3 across all parameters analyzed, exerting beneficial effects on both vascular components starting from lower concentrations. In contrast, MIX-2 exhibited mild efficacy only for some of the parameters analyzed. Notably, both MIX-1 and MIX-3 significantly reduced IL-1β production following 24 h inflammatory stimulation. However, when the stimulus was prolonged up to 48 h, only MIX-1 was able to significantly reduce IL-6 production. This experimental evidence allows us to hypothesize, at least preliminarily, that MIX-1 may exert a beneficial effect in reducing late-onset inflammatory processes following prolonged TMAO stimulation. Accordingly, metabolites present in supernatants derived from *L. rhamnosus* LRH020 and *L. reuteri* PBS072, which compose MIX-1, may display greater efficacy in modulating chronic inflammatory responses in the vascular endothelial component under dysbiotic conditions. In the present study, anti-inflammatory effects were assessed exclusively in endothelial cells, since TMAO-induced inflammation primarily affects the vascular endothelium as an early and physiologically relevant event in the development of vascular dysfunction.

When interpreting the effects of supernatant compositions derived from different probiotic formulations, despite all containing *Lactobacillus* species (as MIX-1 and MIX-3), it is essential to consider the strain specificity [38]. As consistently highlighted in preclinical evidence and meta-analyses, biological activity varies markedly at the strain level, and no generalizable “biotic” effect can be assumed without specifying the strain and specific formulation [39]. Within this framework, the antioxidant efficacy of the individual bacterial strains composing the two most effective formulations, MIX-1 and MIX-3, confirmed distinct differences in the potency of limiting ROS production following a pro-oxidative stimulus in vascular endothelial cells. Specifically, *L. reuteri* PBS072, the only strain shared by both MIX-1 and MIX-3, consistently displayed significant activity and greater potency compared with the other strains tested. *L. rhamnosus* LRH020 in MIX-1 and *L. acidophilus* PBS066 in MIX-3 showed minor efficacy, attenuating ROS accumulation only at the highest concentration examined. In contrast, *L. plantarum* PBS067, present only in MIX-3, did not exert considerable effects under these experimental conditions. Collectively, these findings seem to identify *L. reuteri* PBS072 as the primary contributor to the beneficial antioxidant profile of MIX-1 and MIX-3 and strengthens the critical importance of considering strain specificity when evaluating the functional outcomes of probiotic and probiotic-derived formulations.

*L. reuteri* is a lactic acid bacterium capable of producing metabolites such as tryptophan derivatives, exopolysaccharide, vitamins, reuterin, histamine, short-chain fatty acids (SCFAs), lactic acid, and γ-aminobutyric acid (GABA). Some of these metabolites have been shown to confer protection against acute ischemic cardiac injury in vivo, likely through immunomodulatory and anti-inflammatory mechanisms. Moreover, clinical evidence indicates that *L. reuteri* consumption can significantly reduce total cholesterol, a key cardiovascular risk factor, underscoring potential cardiovascular benefits linked to strain-specific microbial metabolism [40,41].

Furthermore, considering the role of sirtuins in antioxidant processes and cardiovascular aging, the role of this pathway in promoting the antioxidant effects exerted by the more promising formulation MIX-1 was investigated. The results showed that, by blocking the SIRT1 and SIRT2 isoforms (respectively, the isoform most involved in the regulation of endothelial function and the isoform with the greatest antioxidant and anti-inflammatory activity), the antioxidant effect promoted by MIX-1 was significantly attenuated if not completely abolished, suggesting a partial and indirect contribution of SIRT1/2 signaling to the antioxidant effects promoted by MIX-1.

## 4. Materials and Methods

### 4.1. Bacterial Strains and Cell Free Supernatants Production

The probiotic strains were provided by SynBalance S.r.l. (Origgio, VA, Italy). *Lacticaseibacillus rhamnosus* LRH020 (DSM 25568), *Limosilactobacillus reuteri* PBS072 (DSM 25175), *Lactiplantibacillus plantarum* PBS067 (DSM 24937), *Lactobacillus acidophilus* PBS066 (DSM 24936), *Lactobacillus gasseri* LG050 (LMG P-29638), and *Bifidobacterium animalis* subsp. *lactis* BL050 (DSM 25566) were inoculated in De Man, Rogosa, and Sharpe (MRS) broth and incubated overnight at 37 °C under static conditions. Each strain was subsequently inoculated into Dulbecco’s Modified Eagle Medium (DMEM High Glucose, Corning, distributed by Fisher Scientific Italia, Segrate, Milan, Italy) containing 4.5 g/L glucose and L-glutamine, without sodium pyruvate, and supplemented with 10% fetal bovine serum (FBS, CliniSciences, Guidonia Montecelio, Italy), and incubated at 37 °C until reaching the appropriate growth stage. This culture medium is hereafter referred to as HG and was used as vehicle in the in vitro experimental procedures.

Following incubation, bacterial suspensions were centrifuged at 3000 rpm for 15 min. The resulting supernatants were collected and combined at ratio 1:1 or 1:1:1, as follows (Table 2).

Supernatants obtained from individual strains and from the three formulations described above were adjusted to physiological pH and filtered through a 0.22 μm membrane-filter to obtain cell-free supernatants (CFS). These supernatants, obtained by probiotic growth in the eukaryotic medium to allow cytocompatibility, were then employed for in vitro assays using cellular models.

### 4.2. Qualitative/Quantitative Characterization

Samples were analyzed by LC-HRMS to identify metabolites, including lactate, acetate, propionate, and butyrate. The detection limit (DL) allowed detection and quantification only of lactate, whereas the other metabolites were not quantified as their concentrations were below the DL.

Quantitative analysis was performed using a UHPLC Vanquish Flex Binary pump, coupled to a High-resolution Q Exactive Plus Mass Spectrometer, equipped with an ESI source (Thermo Fischer Scientific Inc., Bremem, Germany). The separation was carried out on Kinetex^®^ Biphenyl column (Phenomenex^®^, Castel Maggiore, Bologna, Italy) (100 × 2.1 mm^2^, 2.6 μm particle size). The mobile Phase consisted of 0.1% formic acid in ultrapure water (A) and 0.1% formic acid in ultrapure methanol (B) (all ultrapure solvents were purchased from Romil Ltd. Pure Chemistry, Cambridge, UK) and was delivered at a flow rate of 0.3 mL/min using the following gradient program: 0% (B) from 0 to 2 min, 0–100% (B) from 2 to 27 min, isocratic 100% B for 3 min, return to initial conditions in 1 min, followed by a3-min re-equilibration. The injection volume was 5 μL, and the column oven temperature was set at 30 °C. High resolution MS full scans were acquired on a mass range from 50 to 750 Da with resolution set at 70,000 (at *m*/*z* = 200).

Calibration curves were generated using peak areas obtained from extracted ion chromatograms (EICs), applying a mass extraction window centered on the theoretical *m*/*z* ± 5 ppm. Analyses were performed in negative ionization mode for lactate and in positive ionization mode for the other carboxylates, according to the ionization conditions that provided the highest sensitivity.

Lactate solutions were prepared from a 5 mg/L stock solution, with a calibration curve ranging from 50 µg/mL to 2 mg/mL. For the other carboxylates, a 1 mg/mL stock solution was used, and the calibration curves ranged from 5 µg/mL to 1 mg/mL.

Working solutions were prepared by dilution in water/methanol and were stored at −20 °C. The resulting calibration curves showed excellent linearity, with an R^2^ of 0.99 and a maximum standard deviation below 8%.

LC-HRMS data acquisition and peak integration were performed using Xcalibur software (version 4.8) (Thermo Fisher Scientific, Waltham, MA, USA). Quantification was based on peak area analysis using Microsoft^®^ Office Excel (Microsoft 365) and results were expressed as mg/mL ± standard deviation.

### 4.3. Cell Cultures

The following in vitro experiments were performed using both human aortic smooth muscle cells (HASMC) (Thermo Fisher Scientific, Waltham, MA, USA) and human aortic endothelial cells (HAEC) (Lonza Bioscience, Basel, Switzerland) obtained from different donors. Cells were maintained under optimal proliferative conditions (37 °C, 5% CO_2_) and cultured in T-75 flasks. Cells at 90% confluence and between passages 4 and 10 were used for the experimental procedures. The culture medium for HASMCs (CM-SMC) consisted of basal medium (Medium 231; Thermo Fisher Scientific, Waltham, MA, USA) supplemented with Smooth Muscle Growth Supplement (SMGS; Thermo Fisher Scientific, Waltham, MA, USA) and antibiotics (100 µg/mL streptomycin and 100 U/mL penicillin; Merck KGaA, Darmstadt, Germany). The culture medium for HAECs (CM-EC) consisted of basal medium (EBM^®^-2 Basal Medium; Lonza Bioscience, Basel, Switzerland) supplemented with antibiotics (100 µg/mL streptomycin and 100 U/mL penicillin) and EGM^®^-2 SingleQuots Supplements (Lonza Bioscience, Basel, Switzerland) as required for growth endothelial cell growth.

All experiments on HAECs were performed using two independent cell batches derived from the same donor, selected based on the absence of specific pathologies that could introduce confounding factors. Replicates therefore reflect both biological and technical variability within a single-donor background, and donor-to-donor variability was not included in the statistical analysis.

HASMCs were purchased without selection from specific donors.

Cell cultures were treated with different concentrations of the postbiotic formulations selected based on quantitative estimations of lactate. Except for safety profile assessment, in which postbiotic formulation concentrations ranged from 0.3% to 20% (*v*/*v*), cell-free supernatants were tested at concentrations of 3%, 5%, 10%, and 20% (*v*/*v*) in all experimental procedures. Specifically, as lactate concentrations in the undiluted formulations ranged between approximately 30 and 45 mM, the applied dilutions were calculated to achieve final lactate concentrations overlapping with the 2–10 mM range, which has been reported to exert biologically relevant effects on endothelial cells [42,43,44].

### 4.4. Cell Viability (Cytotoxicity and Pro-Oxidative Stimulation)

Cells were grown to 90% confluence and seeded into clear 96-well plates (cell density: 10^4^ cells/well for both HASMCs and HAECs). After 24 h, the culture medium was replaced with fresh medium, and cells were treated for 24 h with control (CM-SMC for HASMCs and CM-EC for HAECs), vehicle (HG 0.3%, 1%, 3%, 5%, 10%, 20%), or MIX-1, or MIX-2 or MIX-3 (0.3%, 1%, 3%, 5%, 10%, 20% for each mix tested). At the end of the treatment, water-soluble tetrazolium salt-1 (WST-1; Roche, Basel, Switzerland) was added to each well (1:10) and the plate was incubated for 1 h at 37 °C in a 5% CO_2_ incubator. Cell viability was assessed using an EnSpire microplate reader (PerkinElmer, Waltham, MA, USA) at 495 nm. Cytotoxic effects were assessed by normalizing the results obtained from each mix to 100% cell viability, defined as the value obtained after incubation with vehicle alone. After confirming the absence of cytotoxicity of the vehicle alone at the different concentrations tested (HG 0.3%, 1%, 3%, 5%, 10%, 20%) in both cell lines, each concentration of the analyzed mixes was compared with the corresponding concentration of vehicle.

The same experimental procedure was used to evaluate the protective effect of the tested formulations against a pro-oxidative stimulus (H_2_O_2_). Cells were seeded following the procedures and quantities described above. After 24 h, the culture medium was replaced with fresh medium and cells were treated with vehicle (20% HG), or MIX-1, or MIX-2, or MIX-3 (3%, 5%, 10%, 20% for each postbiotic combination tested). After 1 h, H_2_O_2_ 200 μM was added to both HASMC and HAEC cultures. Cell viability was analyzed 2 h after the incubation of the pro-oxidative stimulus using the experimental procedures described above. Results obtained from H_2_O_2_ alone and from preincubation with each tested composition were normalized to 100% cell viability, defined as the value obtained after incubation with vehicle alone.

### 4.5. ROS Production

H_2_O_2_ was used under the same conditions described above to stimulate the production of ROS. In this experiment, HASMCs and HAECs were seeded into black clear flat-bottom 96-well plates (cell density: 3 × 10^4^ cells/well). An aqueous solution of gelatin from porcine skin (Merck KGaA, Darmstadt, Germany) was used as pre-coating, before seeding, to promote cell adhesion to the plate bottom. After 24 h, the culture medium was replaced with fresh medium and the cells were treated with vehicle (20% HG), MIX-1, MIX-2, or MIX-3 (3%, 5%, 10%, 20% for each formulation tested), individual strains included in MIX-1 (PBS072, LRH20) and MIX-3 (PBS072, PBS067, PBS066) at two different selected concentrations (5% and 10% for each supernatant tested). One hour later, H_2_O_2_ (200 μM) was added to both HASMC and HAEC cells. The fluorescent probe dihydroethidium (DHE; Merck KGaA, Darmstadt, Germany) was used to measure the intracellular ROS production. 2 h after the pro-oxidative stimulus, cells were incubated with DHE (10 µM × well) in the dark for 30 min at 37 °C in a CO_2_ (5%) incubator. Fluorescence was detected at λex = 500 nm and λem = 580 nm using the EnSpire microplate reader (PerkinElmer, Waltham, MA, USA).

Furthermore, to evaluate the possible involvement of the sirtuin pathway in ROS production, cells were pre-incubated with sirtinol (1 µM, Cayman Chemical, Ann Arbor, MI, USA), a SIRT1 and SIRT2 inhibitor, for 1 h before the incubation of the selected mix, followed by all experimental procedures described above. This further analysis was carried out exclusively for MIX-1 at concentrations of 5% and 10%, which were selected as the most effective mix and concentrations based on the results of the other tests performed.

### 4.6. Anti-Inflammatory Potential

The anti-inflammatory capacity of the formulations identified as the most effective in the previously performed assays (MIX-1 and MIX-3), at the selected concentrations (5% and 10%), was evaluated by assessing their ability to limit the production of pro-inflammatory cytokines (IL-1β and IL-6) induced by TMAO 600 µM (Merck KGaA, Darmstadt, Germany) in HAECs. Cells were seeded into clear 24-well plates (cell density of 2 × 10^5^ cells/well). TMAO exposure was carried out for 24 h (for the evaluation of IL-1β) and 48 h (for the evaluation of IL-6). MIX-1 and MIX-3 at concentrations of 5% and 10% were pre-incubated for 1 h before the addition of TMAO and were maintained in the medicated culture medium for the entire TMAO exposure period. Subsequently, cells were lysed using a 1% Triton X-100 solution (Merck KGaA, Darmstadt, Germany). Cell lysates were centrifuged at 8000× *g* for 10 min and the resulting supernatants were used for the quantitative determination of IL-1β or IL-6 levels using ELISA kits (Thermo Fisher Scientific, Waltham, MA, USA), according to the manufacturer’s instructions.

### 4.7. Data and Statistical Analysis

Outliers, when present, were excluded from the statistical analysis following identification using the ROUT test (Q = 0.5). The normal distribution of the dataset was assessed using the Shapiro–Wilk test. Data are expressed as mean ± standard error (SEM). One-way ANOVA, followed by Bonferroni’s post hoc test, was performed as statistical analysis to compare the effect of the pro-oxidative stimulus (H_2_O_2_) or the pro-inflammatory stimulus (TMAO) with the vehicle alone and to evaluate the effect of the three formulations at the different concentrations tested. Statistically significant differences were defined as *p* < 0.05. Levels of statistical significance are indicated by the symbols * and §, where one symbol corresponds to *p* ≤ 0.05, two symbols to *p* ≤ 0.01, three symbols to *p* ≤ 0.001, and four symbols to *p* ≤ 0.0001; the specific meaning of each symbol is detailed in the corresponding figure captions. Statistical analyses were performed using GraphPad Prism software (version 8.0.2).

## 5. Conclusions

In conclusion, this study provides novel preclinical evidence supporting the role of probiotic-derived metabolites in modulating the gut–vascular axis under dysbiotic conditions. All three postbiotic formulations evaluated exhibited interesting efficacy profiles in the management of vascular health. Among the tested formulations, MIX-1 composed of *L. rhamnosus* LRH020 and *L. reuteri* PBS072 emerged as the most effective in improving vascular cell functionality, reducing oxidative stress and inflammatory responses, and preserving endothelial and smooth muscle cell integrity, with significant efficacy observed even at lower concentrations. The partial involvement of the sirtuin pathway in the antioxidant response further strengthens the mechanistic relevance of these findings and opens new perspectives for targeted interventions. Furthermore, these findings suggest that, despite the three postbiotic formulations displaying qualitatively comparable metabolic profiles, quantitative differences in lactate production may partially account for the greater efficacy and potency observed for MIX-1 and MIX-3 compared with MIX-2. Furthermore, the antioxidant assessment of the individual strain-derived supernatants that constitute MIX-1 and MIX-3 allowed us to attribute the superior vascular effects of MIX-1, at least in part, to strain-specific properties of the *L. rhamnosus* LRH020 and *L. reuteri* PBS072 included. This finding underscores that accessory genes or distinct enzymatic systems present only in specific strains may drive differences in biological efficacy, even when their qualitative and quantitative metabolic signatures appear largely comparable. Overall, these results suggest that selected probiotic-derived postbiotic formulations may represent promising candidates for innovative adjuvant strategies aimed at restoring vascular homeostasis in the context of dysbiosis, thereby paving the way for further in vivo validation and translational investigation.

Some limitations of the present study should be acknowledged. The experiments were conducted directly on vascular endothelial and smooth muscle cells and therefore do not fully reflect the complexity of in vivo conditions. In particular, the precise identity and concentrations of microbial metabolites that effectively reach the vascular compartment remain to be elucidated. Moreover, factors such as gut barrier integrity, the degree of dysbiosis, and potential alterations in metabolite pharmacokinetics under physiological or pathological conditions, including hypertension, atherosclerosis, or endothelial dysfunction, were not addressed in the present work. In addition, although lactate levels appear to play a role in the beneficial effects observed when applied at concentrations comparable to physiological and transiently elevated conditions, their actual contribution to the overall biological activity remains to be fully elucidated. Accordingly, future studies using lactate-matched controls or lactate-depletion strategies will be essential to establish a causal link between lactate levels and the enhanced efficacy of MIX-1 and MIX-3. Finally, the assessment of sirtuin pathway involvement using sirtinol represents an indirect experimental approach and is limited by incomplete isoform specificity and the possibility of off-target effects. Future investigations should also aim to characterize the most promising metabolite mixtures in more advanced experimental settings, including ex vivo isolated vessel systems and in vivo murine models, to better elucidate their mechanisms of action and translational relevance in maintaining vascular health. In addition, the use of more selective pharmacological tools and/or genetic approaches (e.g., siRNA-mediated knockdown or overexpression strategies) will be necessary to definitively establish the role of specific sirtuin isoforms. Finally, although endothelial-derived inflammatory mediators may secondarily affect vascular smooth muscle cells, the evaluation of inflammatory responses in smooth muscle cells should be addressed in future studies.

## Figures and Tables

**Table 1:** LC-HRMS quantitative analysis of lactate in MIX-1, MIX-2, and MIX-3. Statistical significance versus MIX-2 (** *p* < 0.01).

	MIX-1		MIX-2		MIX-3	
	mg/mL	SEM	mg/mL	SEM	mg/mL	SEM
Lactate	4.04 **	0.24	2.71	0.15	3.35	0.31

**Table 2:** Bacterial information about the postbiotic formulations.

	Taxonomy	Strain Code	EU Collection
MIX-1	*Lacticaseibacillus rhamnosus*	LRH020	DSM 25568
	*Limosilactobacillus reuteri*	PBS072	DSM 25175
MIX-2	*Bifidobacterium animalis* subsp. *lactis*	BL050	DSM 25566
	*Lactobacillus gasseri*	LG050	LMG P-29638
MIX-3	*Lactiplantibacillus plantarum*	PBS067	DSM 24937
	*Lactobacillus acidophilus*	PBS066	DSM 24936
	*Limosilactobacillus reuteri*	PBS072	DSM 25175

## Data Availability

Data is contained within the article in terms of individual data highlighted in each plot. Dataset containing the specific numerical data is available on request from the authors.
